# A Cytological Review of Follicular Dendritic Cell-Derived Tumors with Emphasis on Follicular Dendritic Cell Sarcoma and Unicentric Castleman Disease

**DOI:** 10.3390/diagnostics12020406

**Published:** 2022-02-04

**Authors:** José A. Jiménez-Heffernan, Cristina Díaz del Arco, Magdalena Adrados

**Affiliations:** 1 Department of Pathology, University Hospital La Princesa, Diego de León, 62, 28006 Madrid, Spain; magdalena.adrados@salud.madrid.org; 2 Department of Pathology, University Hospital Clínico San Carlos, Profesor Martín Lagos s/n, 28040 Madrid, Spain; cristina.diaz@salud.madrid.org

**Keywords:** Castleman disease, cytology, fine needle aspiration, follicular dendritic cell, follicular dendritic cell sarcoma

## Abstract

Follicular dendritic cells (FDCs) are antigen-presenting cells located in the germinal centers of the lymph nodes. Among the few tumors showing FDC differentiation are follicular dendritic cell sarcoma (FDCS) and Castleman disease (CD), more precisely the unicentric hyaline vascular (HV) variant. Both are relatively rare tumors, and the diagnostic cytological experience is limited to descriptions of isolated cases or small series. The purpose of this review is to bring together all the available cytological published information, and our personal experience, in order to obtain a global idea of the cytological features of these peculiar FDC-derived tumors. The different descriptions of FDCS are very similar, reflecting a tumor that shows repetitive and characteristic cytological features. It shows a dimorphic population of mature lymphocytes and large tumoral cells with partial spindle morphology. Most cases of HV variant of CD can be recognized as benign upon cytology, however a precise diagnosis seems more difficult. It is characterized by reactive lymphocytes mixed with vessels and FDCs, either single or forming syncytial aggregates. Both, FDCS and CD are challenging for cytological diagnosis in which a high index of suspicion is necessary for a correct preoperative assessment. Cytology is very useful for follow-up of recurrences and metastases.

## 1. Introduction

Follicular dendritic cells (FDCs) are antigen-presenting cells located in the germinal centers of the lymph nodes [1,2,3]. Maximow originally described them as “reticular cells” in 1927 and noted the propensity of their cytoplasmic processes to form a network [4]. Their normal function in antigen presentation is known in detail since they play a crucial role in the maturation of B cells. They seem to have an important role in many reactive and lymphoid neoplastic disorders [3]. FDCs are abundant in reactive nodes and in the microenvironment of some lymphomas. In follicular lymphomas, FDC-dependent signaling promotes B cell proliferation as well as angiogenesis and migration. Their loss seems to have importance in the malignant progression of mantle zone lymphoma, marginal zone lymphoma, and Hodgkin’s lymphoma (HL). Similarly, they seem to participate in advanced stages of angioimmunoblastic T-cell lymphoma where an abnormal hyperplastic meshwork of FDCs in extrafollicular areas is often present [3]. Interestingly, few tumors show FDC differentiation. In 1986, Monda et al. described a malignant neoplasm showing features of FDCs [5]. FDC sarcoma (FDCS) is the term used by the World Health Organization [6]. 

A second neoplastic entity that partially derives from FDCs is Castleman disease (CD). Clinically, it is classified as unicentric when one anatomic lymph node is affected and multicentric when multiple lymph node sites are involved. The unicentric hyaline vascular (HV) variant of CD is the most common form and it seems to be a clonal neoplastic process derived from FDCs [7,8,9]. There are limited studies evaluating the cell type responsible for multicentric CD. In addition to FDCs, potential cells include lymphocytes, plasma cells, monocytes, and endothelial cells [8]. A remarkable feature is that both entities, FDCS and CD, are related. It is relatively common to see cases of CD evolve into FDCS, and CD often shows dysplastic FDCs (discussed below). 

From a cytological diagnostic perspective, these different tumoral entities are difficult to diagnose so a precise preoperative recognition is often impossible. These tumors are rare and cytological descriptions are limited to isolated case reports or small series. Therefore, they are considered to be “classic” among cytopathologists since they are always a diagnostic challenge. In this report, we will review the cytological literature and our own experience with FDCS and CD. We will discuss their main morphological features and the possibilities of reaching a precise diagnosis.

## 2. The Normal Follicular Dendritic Cell and Its Role in Disease

FDCs are located in the primary and secondary follicles of the B-cell areas of the lymph nodes, spleen, and mucosal-associated lymphoid tissue. They constitute an exception among hematolymphoid cells since they do not derive from a common bone marrow hematopoietic precursor [1,2,3]. They are stromal in origin and develop from vascular mural cells within the lymph nodes [1,2]. The same stromal cellular origin is shared with fibroblastic reticular cells (FRCs) that are also found in secondary lymphoid organs. These cells play an important structural role, but also contribute as immune response regulators [2]. Similarly, FDCs must be distinguished from interdigitating dendritic cells that derive from the bone marrow and serve as antigen-presenting dendritic cells commonly present in lymph node T-cell areas. Conventional dendritic cells activate naive T cells by presenting a processed antigen via a major histocompatibility complex (MHC) molecules. In contrast, FDCs present an unprocessed antigen in the form of immunocomplexes [1,2,3]. FDCs are non-phagocytic and lack class II MHC molecules. They bind antigen via complement receptors (CD21 and CD35) which attach to the complement associated to immunocomplexes. They locate in B follicles and are specialized in the capture of immunocomplexes that can remain for long periods in the cell surface and be presented to surrounding B-lymphocytes. This function is facilitated by the numerous cytoplasmic prolongations and interdigitations that form an intricate network connected through desmosomes [1,2,3,10]. In collaboration with macrophages, FDCs control the removal of apoptotic germinal center B cells. In addition, they seem to play an important role in maintaining lymphoid follicle microarchitecture since they attract B cells towards the follicles [1,2]. 

FDCs main role in disease relates to the development of autoimmune disorders, mainly rheumatoid arthritis, and systemic lupus erythematosus. They contribute to autoimmunity through the presentation of self-immunocomplexes. FDCs drive the selection and affinity maturation of self-reactive B cells. In this sense, they serve as gatekeepers of tolerance ensuring the rapid removal of dying cells, negatively selecting naive self-reactive B cells [1,2]. The role of FDCs in the progression of lymphomas has already been mentioned [3].

**Figure 1:**
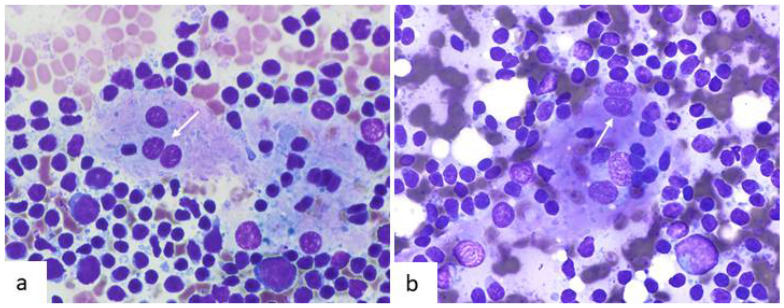
Cytological features of follicular dendritic cells (FDCs) in fine needle aspiration samples of reactive lymph nodes. (**a**,**b**) In both cases, they form a part of germinal center fragments and are mixed with numerous reactive lymphocytes. They are large cells with ill-defined cytoplasm that determines a syncytial appearance. Nuclei are round to slightly oval with occasional binucleation (white arrows) that shows molding tendency (“kissing” pattern) (Diff-Quik, ×600).

Under light microscopy, they are large cells with an abundant ill-defined eosinophilic cytoplasm. Their nuclei are round to ovoid with a small eosinophilic nucleolus and finely dispersed chromatin. Vey often they are binucleated showing overlapping or molding (“kissing” pattern) [10]. The most common markers used in diagnostic immunohistochemistry are CD21, CD23 and CD35. They also express desmoplakins, epidermal growth factor receptor (EGFR), claudin 4 and podoplanins [6,10,11]. On fine needle aspiration (FNA) cytology, FDCs are relatively easy to recognize. They are large cells that are usually seen within germinal center fragments, accompanied by lymphocytes and tingible body macrophages. They can also be seen as single cells. Their cytoplasm is large and ill-defined which creates a syncytial aspect when cells are grouped. No cytoplasmic phagocytic debris or vacuolization are present. Nuclei are round to oval and binucleation is a common feature, resembling the “kissing” pattern described on histology (Figure 1). On Papanicolaou stained smears, the nuclear contour is well defined, they show vesicular chromatin and a small nucleolus.

## 3. Relation between Castleman Disease and Follicular Dendritic Cell Sarcoma

Approximately 10% to 20% of FDCS, most commonly in extranodal sites, are associated to CD, usually the hyaline vascular (HV) variant [11]. In addition to the association of both entities [12,13,14], Chan et al. observed FDC hyperplasia transforming to FDCS by sequentially studying biopsies from the same site in a patient with HV-CD [15]. Some cases of CD preceding FDCS show areas of FDC proliferation, and sarcoma arise in these areas. As in the hyperplasia-dysplasia-neoplasia sequence seen in some carcinomas, FDCS may arise in lymph nodes harboring dysplastic FDCs [15,16,17]. As we will see when analyzing the cytological features of CD, dysplastic FDCs are not an uncommon finding. The significance of these large dysplastic FDCs is still unknown but it may help to explain the relation between CD and FDCS. In fact, one of the first publications describing the cytological features of two patients with FDCS appeared in 1997 [18]. One of the two patients reported had a history of HV-CD with histologic evidence of dysplastic FDCs. Some authors consider that the association of CD and FDCS may be underestimated, since FDCS overgrowth may prevent the histologic recognition of residual HV-CD [10]. In addition to other markers, FDCs of CD and FDCS share an intense expression of EGFR that is rarely seen in normal or reactive FDCs [19]. Similarly, they can have a common miRNA profile [20]. Although there are few molecular studies, a remarkable coincidence was the detection of the PDGFRB N666S mutation, that often occurs in HV-CD, in a case of FDCS with a previous history of CD [10]. These findings further strengthen the relationship between these two entities.

## 4. Overview of Follicular Dendritic Cell Sarcoma

Monda, Warnke and Rosai recognized a tumor showing FDC differentiation in 1986 [5]. Subsequent studies confirmed this finding and nowadays FDCS is a well-accepted entity [6]. It has no gender predilection, occurs during adulthood and is rare in children. The most common location is extranodal (liver, spleen, and gastrointestinal tract) [6,10]. Lymph nodes are affected in 20–30% of the cases, with cervical nodes being the most frequently involved. An inflammatory pseudotumor-like variant exists that is more common in women with liver or splenic involvement and systemic symptoms. FDCS shows low to intermediate aggressive behavior with local recurrences and distal metastases in approximately one third of cases, respectively. An early diagnosis is of the greatest importance since survival diminishes considerably with the presence of advanced disease. Histology reveals a spindle cell proliferation with varied architectural patterns, arranged in storiform or whorled fascicles, trabeculae, or diffuse sheets. Lymphocytes are abundant throughout the tumor. Multinucleated cells can be seen. Cell borders are generally indistinct, imparting a syncytial appearance. The cytoplasm is moderately abundant and eosinophilic. The tumor cells have elongated or ovoid nuclei with vesicular or granular chromatin and small nucleoli. Nuclear pseudoinclusions can occasionally be seen [10,11]. Most cases are relatively bland but cytologic atypia may be present. High-grade histologic features are more common in deeply located, recurrent or metastatic lesions and include cellular atypia, numerous mitoses, and necrosis. 

Histological features associated with a worse prognosis include size (≥6 cm), necrosis, high mitotic count (≥5 mitoses per 10 high-power fields), and significant cytological atypia [10]. In the inflammatory pseudotumor-like variant of FDCS, tumor cells can be difficult to recognize since they are interspersed among a prevalent inflammatory infiltrate mainly composed of lymphocytes and plasma cells. Importantly, in FDCS associated with CD a transition from HV-CD with FDC dysplasia to evident tumor proliferation can be observed [6,10,11]. Immunohistochemistry is necessary to establish a diagnosis of FDCS. Neoplastic cells express one or more dendritic cell markers CD21, CD35, CD23, podoplanin, fascin, clusterin. They are negative for CD45, CD34, CD30, CD163, cytokeratin, HMB45, Melan A, CD1a but can express EMA, EGFR, CD68 and S100 [6,10,11].

## 5. Cytological Features of Follicular Dendritic Cell Sarcoma

Reflecting the rarity of FDCS, cytological descriptions of this tumor are limited to case reports. Our review of the cytological literature revealed 24 articles describing 26 patients (Table 1) [18,21,22,23,24,25,26,27,28,29,30,31,32,33,34,35,36,37,38,39,40,41,42,43]. 

After reviewing the cytological articles, it is remarkable to observe that morphological descriptions are very similar. The tumor shows repetitive and characteristic cytological features that are summarized in Table 2. 

**Figure 2:**
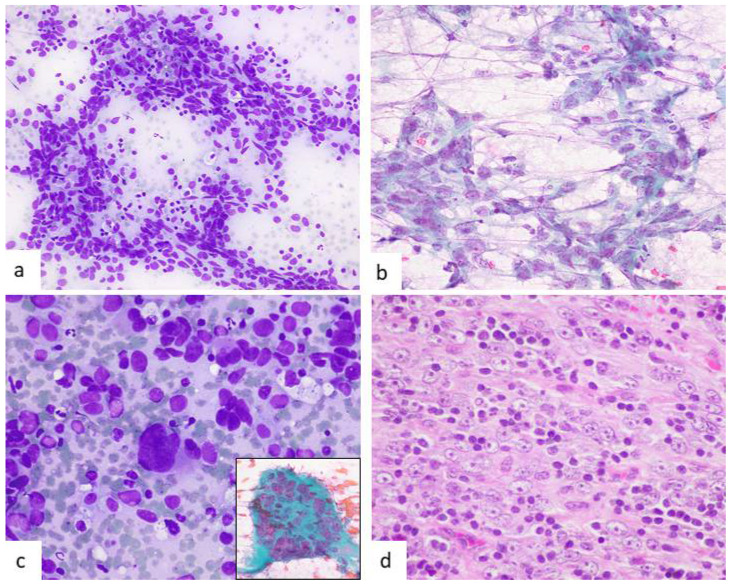
A case of recurrent FDCS. (**a**) Ill-defined aggregates of oval to spindle cells and lymphocytes (Diff-Quik, ×200). (**b**) Long cytoplasmic interconnecting processes create a fibrillary appearance (Papanicolaou, ×400). (**c**) Occasional cells showed nuclear atypia (Diff-Quik, ×400). The inset shows a large multinucleated cell (Papanicolaou, ×600). (**d**) Histological image with large tumoral cells with vesicular nuclei, prominent nucleoli, and numerous lymphocytes (HE, ×400).

The first description made by Dusenbery and Watson in 1997 already highlighted the main clues for diagnosis [21]. Although the authors made an erroneous diagnosis, they mention that “the aspirate smears in this case displayed a constellation of findings that might suggest the correct diagnosis” [21]. This commentary is present in almost all the cytological reports that followed, as if after a retrospective analysis the authors of the different articles realized that histological features were mirrored by cytological ones. Smears are cellular and cells distribute singly or forming irregular, poorly cohesive aggregates. Cytology reveals a characteristic dual cell population (dimorphic pattern): firstly, reactive small lymphocytes and plasma cells with accompanying lymphoglandular bodies, that are an important clue for diagnosis. The second population corresponds to neoplastic FDCs that can have variable morphology. They are large cells with ill-defined cytoplasm and round to oval nuclei that can show bi-multinucleation. Although tumoral cells can be in contact and form irregular groups, the impression is not that of a conventional cohesive neoplasm, and this feature is an important clue for FDCS recognition. Their shape is variable, but most reports describe them as oval to spindle with some showing epithelioid or polygonal morphology. Different authors have made an emphasis on long, slender cytoplasmic processes (Figure 2b) variably described as stellate cells [23], interwoven dendritic (spider web-like) [30], or interconnecting [36]. Probably, the most remarkable description was that made by the group of Pambuccian [36] that mentions such cytoplasmic meshwork as resembling the head of the mythical monster Medusa. Shorter ones created a starfish-like appearance. Mitotic activity and necrosis are more common in recurrent or metastatic cases (Figure 2). 

**Figure 3:**
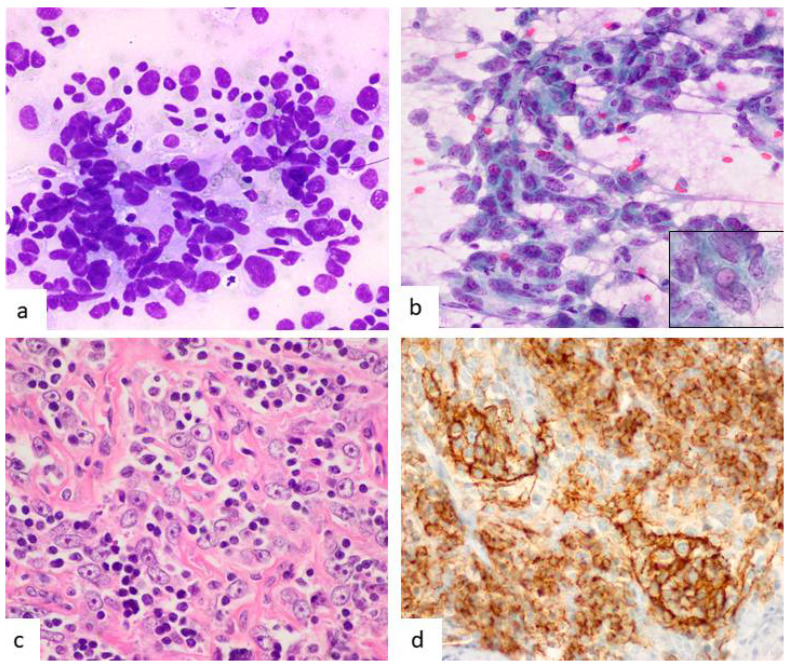
A case of follicular dendritic cell sarcoma in a bronchial lymph node. (**a**) Irregular clusters and single pleomorphic large tumoral cells with lymphocytes. Most cells have oval nuclei (Diff-Quik, ×400). (**b**) Cells showed slender, elongated cytoplasmic processes and spindle cell morphology. The inset shows a nuclear pseudoinclusion (both Papanicolaou, ×400). (**c**) Histology is characteristic with a dimorphic cell pattern of lymphocytes and large tumoral cells with oval nuclei and evident nucleoli (HE, ×400). (**d**) Neoplastic cells showing intense CD35 expression (immunoperoxidase, ×400).

Noting the tendency to a spindle morphology is extremely important since it is rare in carcinomas (Figure 2 and Figure 3). The clusters are admixed with lymphoid cells that often are superimposed, showing no emperipolesis. The nuclei tend to have an oval morphology with finely granular or vesicular chromatin. A variable sized nucleolus is usually present. When binucleated cells have a large nucleolus, they can resemble Reed–Sternberg cells. Other characteristic findings are nuclear grooves and sometimes nuclear pseudoinclusions (Figure 3b). 

Concerning FDCS, we have personal experience with four patients, one of which was previously published [25]. Two corresponded to cervical nodal recurrences and a third one to a soft tissue metastasis. A fourth case presented as a pulmonary mass, and carcinoma was clinically suspected (Figure 3). The tumor showed no expression of the usual lung immunohistochemical markers and was diagnosed as a large cell carcinoma. After surgery, the lobectomy sample revealed FDCS in a bronchial lymph node. This case showed the aforementioned cytological features (Table 2) and the main reason for misdiagnosis was its misleading clinical presentation as a pulmonary carcinoma.

The cytological differential diagnosis of FDCS has been extensively discussed in previous reports and partially depends on tumor location. Fortunately, the tumor has a worrisome aspect so even in the case of a non-specific or erroneous cytological diagnosis there is usually no relevant delay in reaching a final pathologic diagnosis since a biopsy will follow. The most important considerations are carcinomas with a lymphoid stroma, especially metastatic nasopharyngeal carcinoma (in cervical cases), thymoma (in mediastinal cases) mesenchymal tumors of the lymph nodes and inflammatory myofibroblastic tumor, gastrointestinal stromal tumors, and other sarcomas in abdominal cases. Ectopic meningioma and malignant melanoma are usually devoid of abundant lymphocytes. Interdigitating dendritic cell sarcoma and the rare fibroblastic reticular cell tumor can also resemble FDCS. Other important diagnostic considerations are the rare spindle cell variant of large cell lymphoma and sarcomatoid anaplastic large cell lymphoma [44,45]. The accumulated cytological experience with FDCS permits us to conclude that it is a tumor with repetitive morphologic features. A preoperative recognition is possible, but it requires a high level of suspicion from the pathologist. Immunocytochemistry can confirm the diagnosis, but due to its rarity and the necessity to evaluate histological variables related to malignancy, total excision and complete pathological study is mandatory. 

## 6. Overview of Castleman Disease

The very recent classification of CD into four disorders is based on clinicopathologic criteria [7] and obviously is not reflected in the previous cytological literature. CD includes unicentric CD (UCD) and multicentric CD (MCD), the latter of which is divided into idiopathic (iMCD), human herpes virus-8 (HHV8)-related MCD (HHV-8 MCD) and polyneuropathy, organomegaly, endocrinopathy, monoclonal plasma cell disorder, and skin changes (POEMS)-associated MCD (POEMS-MCD). Previous classifications divided CD into two extreme types, the HV and plasma cell (PC) variants, with an intermediate mixed type. In 2017 an expert panel changed the terminology into two main histopathologic variants and a third one showing mixed features [46]. Firstly, in one end of the spectrum, the HV (or hypervascular) CD that often shows regressed germinal centers and prominent vascularization. They introduced the term hypervascular to avoid the association of HV variant with UCD, since it can also be present in MCD. Secondly, the plasmacytic variant has hyperplastic germinal centers with prominent plasmacytosis. This variant is more common in HHV-8-MCD, iMCD and POEMS-MCD. Lymph nodes with mixed histopathology show both HV and PC features. It can be observed in UCD and iMCD and nodes show sheet-like plasmacytosis and numerous regressed germinal centers. 

As mentioned in the introduction, it seems clear that UCD derives from FDCs, while in multicentric cases the cellular source is not so evident. UCD is the most common clinical form of CD and the majority of reported cytological cases of CD correspond to this variant. Therefore, we will mention some other relevant histological features [7,9,46]. The nodes tend to show capsular fibrosis with fibrous bands traversing through their parenchyma. Lymphoid follicles are numerous, with abnormal-appearing regressed germinal centers. These show a marked reduction in lymphocytes and a prominence of FDCs. The appearance of the follicles in this variant is at least partially due to expanded and disrupted FDC networks. The presence of giant “dysplastic” FDCs within both the germinal centers and mantle zones is another relevant finding. They appear as large cells with indistinct cytoplasm or naked nuclei with irregular nuclear margins and variable sized nucleoli. In some cases, the cytoplasm appears to contain small lymphocytes. As in FDCS, FDCs may be bi- or multinucleated. There exists a marked proliferation of small blood vessels within the interfollicular zones with characteristic hyalinized and thickened walls. In addition, there is an expansion of the mantle zone with numerous concentric rings of small lymphocytes (“onion skinning” pattern) surrounding the regressed germinal centers. It is typical to see one or more venules penetrating through this thickened mantle zone towards the germinal center, forming the so-called “lollipop” follicles. Lymph nodes also show an absence of sinuses. A stromal-rich variant of HV-CD exists. It is characterized by a marked ‘angiomyoid’ interfollicular stromal proliferation composed of hyperplastic spindle cells and blood vessels [9,16].

## 7. Cytological Features of Castleman Disease

As one can expect from the histological descriptions of HV and PC variants of CD, the cytomorphology of both entities differs considerably. As we will see, a cytological diagnosis of CD is difficult. Even if suspected, a confirmatory biopsy will be necessary since it is required for the clinical management of patients [7,46]. Flow cytometry is non-specific but helps to exclude lymphoma. Cytological descriptions on CD are almost limited to isolated case reports and small series. Most of them report unicentric cases of the HV variant (Table 3) [47,48,49,50,51,52,53,54,55,56,57,58,59,60,61,62,63,64,65,66,67,68,69].

Almost all of them coincide in that a specific cytological diagnosis is challenging. The first report was published in 1982, and since then at least 23 articles including 40 patients have followed [47,48,49,50,51,52,53,54,55,56,57,58,59,60,61,62,63,64,65,66,67,68,69]. Thirty-two correspond to the HV type; six to the PC variant, and two are mixed or not specified. Table 4 summarizes the main cytological features of the HV variant of CD. There are three main clues for the diagnosis: (1) the presence of numerous small lymphocytes, as single cells or forming aggregates with FDCs; (2) a second population of large FDCs with variable atypia; and (3) vessels traversing lymphocytic aggregates or as single fragments, with or without wall hyalinization. Smears are hypercellular and consist of an abundant population of small lymphocytes that are in close association to FDCs, which can be arranged in cohesive fragments. Such fragments can show traversing capillaries, occasionally with hyalinized walls. In a POEMS-related case, Owen et al. reported almost intact follicular structures with vague concentric layers of lymphocytes, reminiscent of the “onion skinning” arrangement seen on histology [55]. Vessels are identified as isolated elongated or branching structures. The diagnostic importance of vessels was already mentioned in the first cytological description [47] and is highlighted by most authors [50,55,56,57,58,59,61,66,67,68,69]. Reactive germinal centers are rare but possible, so their presence does not exclude the diagnosis. Similarly, tingible body macrophages can be found in some cases.

**Figure 4:**
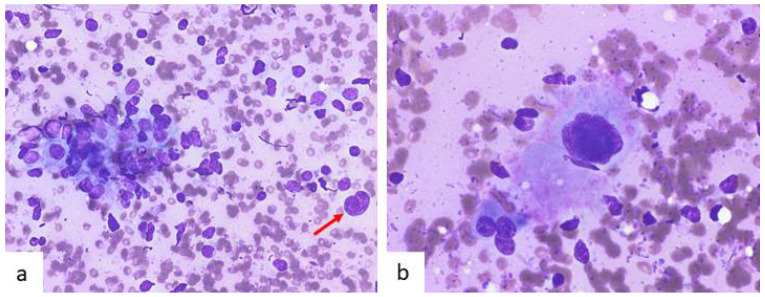
Cytological images in case of HV-CD. (**a**) The image shows an aggregate of FDCs (left) as well as a large single one with no cytoplasm and a nuclear indentation (red arrow) (Diff-Quik, ×200). (**b**) Some FDCs are dysplastic with very large size and irregular nuclear contours (Diff-Quik, ×600).

In addition to lymphocytes, a “second” population of large cells is present. This second population corresponds to FDCs that can distribute as single cells or forming small clusters with a syncytial morphology (Figure 4). FDCs show ample cytoplasm with indistinct cell borders and occasional emperipolesis [54,66,67]. 

Most nuclei have regular borders with fine chromatin, small nucleoli and occasional binucleation. Hidvegui et al. described a peculiar coarse chromatin (“wrinkled tissue paper”) [47] that has been rarely reported by others [67,68]. Nucleoli sometimes can be large, and nuclear grooves and indentations can be prominent (Figure 4). Large binucleated cells with relevant nucleoli may be misinterpreted as Reed–Sternberg cells [53,56,60]. In fact, HL is one of the main pitfalls in the cytological diagnosis of CD. An extreme form of FDC change are the so-called giant “dysplastic” cells. They are also present in the PC variant of CD. At least one cytological report described them [50]. Smears rarely show neutrophils, granulomas, or eosinophils.

Concerning the differential diagnosis of HV-CD, we must remember that most of the affected nodes are large and show an abnormal ultrasonographic image. Therefore, we suggest always including HV-CD in the list of non-malignant lymph node disorders that can present as a pathological adenopathy. Reactive lymphoid hyperplasia with follicular hyperplasia will normally show evident germinal centers and tingible body macrophages. Vessels can be present mainly if the needles used for aspiration are thick. Such nodes rarely exceed 3 cm in size. Among lymphomas, HL and angioimmunoblastic T-cell lymphoma (AITL) deserve a special mention. Large FDCs with dysplastic features may resemble Hodgkin’s related neoplastic cells, especially when binucleated. Macronucleoli is absent in CD as well as the reactive background of HL that usually include eosinophils. The regressed follicles, vascular and FDC proliferation so characteristic of AITL can resemble CD [70]. On cytology, AITL show fragments of lymphoid tissue with a prominent vascular network and FDC aggregates admixed with lymphocytes, and no tingible body macrophages. These structures have been named “dendritic cell-lymphocyte complexes” and can resemble what is seen in HV-CD [70,71,72]. Since atypia may not be relevant in low-grade lesions, smears may look benign. Flow cytometry can be a helpful aid [72]. 

**Figure 5:**
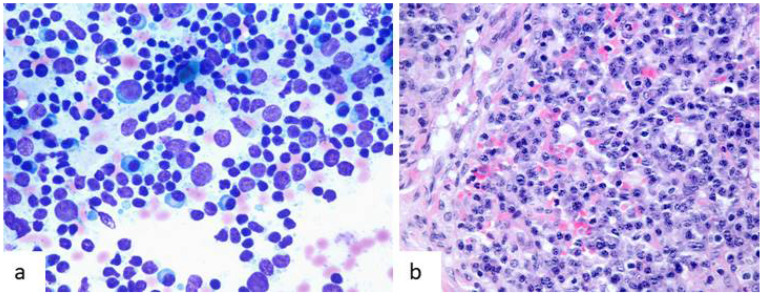
Cytological images in a case of plasma cell Castleman disease (PC-CD). (**a**) Smears show a reactive image with lymphoid polymorphic cells accompanied by numerous mature plasma cells (Diff-Quik, ×400). (**b**) Lymph node histology reveals areas of sheet-like distribution of plasma cells (HE, ×400).

Cytological descriptions of PC-CD are limited to six patients [49,63,65]. We previously published our experience with three cases [65]. Findings were those of a reactive lymph node cell population with a significant number of accompanying mature plasma cells (Figure 5). Dendritic cells and tingible body macrophages from germinal centers were also present. PC-CD is associated to HHV-8-MCD, often in the setting of human immunodeficiency virus infection, and POEMS-MCD. In these precise contexts, FNA can be extremely helpful since plasmacytosis may suggest the diagnosis of PC-CD. 

## Figures and Tables

**Table 1:** Reported cytological studies of follicular dendritic cell sarcoma.

Authors	n	Tumor Site	Original Diagnosis	Other
Dusenbery and Watson [21]	1	Cervical node	Carcinoma	Thyroid involvement
Wright et al. [18]	2	Cervical nodes	Malignant tumor	One case associated to HV-CD
Ryley et al. [22]	1	Axillary node	Metastatic carcinoma or sarcoma	Contralateral axillary involvement
Herceg et al. [23]	1	Axillary nodes	Recurrent FDCS	Scrape cytology
Guiter et al. [24]	1	Cervical node	Malignant mesenchymal tumor	Cell block available
Vicandi et al. [25]	1	Cervical node	Recurrent FDCS	-
Gaffney et al. [26]	1	Abdominal with metastases	Metastatic FDCS (lung)	Poor clinical response
Loo et al. [27]	1	Abdominal	Carcinoma	Previous colonic carcinoma
Mohanty et al. [28]	1	Inguinal node	HL/melanoma	Bone marrow involvement
Ren et al. [29]	1	Spleen	Metastatic FDCS (liver)	-
Yang et al. [30]	1	Abdominal	Metastatic FDCS (liver)	-
Fan et al. [31]	2	Cervical node/nasopharyngx	Atypical/Recurrent FDCS	One case associated to HV-CD
Granados et al. [32]	1	Liver	Not mentioned	IPT-like variant, imprint sample
Tokyol et al. [33]	1	Cervical node	Malignant tumor, FDCS suggested	Recurrence two years later
Song et al. [34]	1	Abdominal node	Lymphoma	Imprint sample
Kure et al. [35]	1	Cervical node	Neuroendocrine tumor	HIV patient
Czapla et al. [36]	1	Cervical node	FDCS	Cell block available
Hang et al. [37]	1	Spleen	Atypical, cannot exclude HL	IPT-like variant
Ojha et al. [38]	1	Cervical node	FDCS	-
Dutta et al. [39]	1	Cervical node	Malignant tumor (carcinoma)	Cell blok available
Abdou et al. [40]	1	Cervical node	Carcinoma	Associated HV-CD
Walke et al. [41]	1	Cervical node	Not mentioned	Cystic component
Asiry et al. [42]	1	Cervical node	Malignant neoplasm	Hypocellular cell block
Xia et al. [43]	1	Cervical node	FDCS	Cell block available

Abbreviations: n—number of cases, HV-CD—hyaline vascular Castleman disease, FDCS—follicular dendritic cell sarcoma, HL—Hodgkin´s lymphoma, IPT—inflammatory pseudotumor, HIV—human immunodeficiency virus.

**Table 2:** Main cytological features of follicular dendritic cell sarcoma.

Hypercellular Smears
Dimorphic cell population Small lymphocytes and plasma cells Large tumoral follicular dendritic cells
Loosely cohesive or syncytial tumor aggregates and single cells
Tumoral cells with variable morphology (polygonal and spindle) Evident cytoplasm with occasional elongated interconnecting processes Bi-multinucleation with occasional Reed-Sternberg like cells
Oval to round nuclei Finely granular to vesicular chromatin Variable size nucleoli Nuclear grooves and occasional pseudoinclusions
Mitoses, atypia, and necrosis more common in metastatic and recurrent cases

**Table 3:** Reported cytological studies of Castleman disease.

Authors	n	Histologic Variant	Type of Sample	Original Diagnosis
Hidvegui et al. [47]	1	HV	FNA	Consistent with CD
Sterret et al. [48]	1	Probably HV	FNA	Benign
Stanley et al. [49]	1	Multicentric PC	CSF	Benign
Chan and McGuire [50]	1	HV	FNA	Benign
Cangiarella et al. [51]	1	Not mentioned	FNA	Inconclusive
Panayiotides et al. [52]	1	HV	FNA	Lymphoma cannot be excluded
Meyer et al. [53]	2	HV	FNA	Benign, HL cannot be excluded
Taylor and Smeeton [54]	1	HV	FNA	Inconclusive
Owens et al. [55]	1	HV	FNA	Benign
Mallik et al. [56]	3	HV	FNA	Atypical (2) and HL (1)
Deschenes et al. [57]	1	HV	FNA	Atypical
Nanda et al. [58]	1	HV	FNA with cell block	Benign, CD
Sudha et al. [59]	2	HV	FNA	Benign, CD
Naik et al. [60]	1	HV	FNA	HL cannot be excluded
Gohsh et al. [61]	5	HV	FNA	Benign
Kashab et al. [62]	1	HV	EUS-FNA with cell block	Benign, CD
Lobo et al. [63]	1	PC	Effusion	Benign
Gill et al. [64]	1	HV	FNA	Benign, CD
Gordillo-Velez et al. [65]	3	PC	FNA	Benign
Malzone et al. [66]	1	HV	FNA	Atypical, consider CD
Murro et al. [67]	8	HV (5), PC (2), mixed (1)	FNA (2), touch preps (6)	Non HL (1), HL (1)
Harries et al. [68]	1	HV	FNA with cell block	Benign, CD
Singh et al. [69]	1	HV	FNA	Benign, granulomatous

Abbreviations: n—number of cases, FNA—fine needle aspiration, HV—hyaline vascular, PC—plasma cell, CSF—cerebrospinal fluid, CD—Castleman disease, HL—Hodgkin´s lymphoma, FDC—follicular dendritic cell.

**Table 4:** Main cytological features of hyaline vascular (hypervascular) Castleman disease.

Hypercellular smears with a predominance of small lymphocytes
Tissue fragments with vessels
Single or small clusters of dendritic cells
Dendritic cell variants (dysplastic)
Rare germinal centers or tingible body macrophages
Residual germinal centers penetrated by capillaries
Capillary fragments (sometimes hyalinized)
